# Extrapolative Capability of Two Models That Estimating Soil Water Retention Curve between Saturation and Oven Dryness

**DOI:** 10.1371/journal.pone.0113518

**Published:** 2014-12-02

**Authors:** Sen Lu, Tusheng Ren, Yili Lu, Ping Meng, Shiyou Sun

**Affiliations:** 1 Key Laboratory of Tree Breeding and Cultivation of the State Forestry Administration, Research Institute of Forestry, Chinese Academy of Forestry, Beijing 100091, China; 2 Collaborative Innovation Center of Sustaintable Forestry in Southern China, Nanjing Forestry University, Nanjing 210037, China; 3 Department of Soil and Water, China Agricultural University, Beijing 100193, China; 4 Institute of Agro-resources and Environment, Hebei Academy of Agriculture and Forestry Sciences, Shijiazhuang 050051, China; Institute for Sustainable Plant Protection, C.N.R., Italy

## Abstract

Accurate estimation of soil water retention curve (SWRC) at the dry region is required to describe the relation between soil water content and matric suction from saturation to oven dryness. In this study, the extrapolative capability of two models for predicting the complete SWRC from limited ranges of soil water retention data was evaluated. When the model parameters were obtained from SWRC data in the 0–1500 kPa range, the FX model (Fredlund and Xing, 1994) estimations agreed well with measurements from saturation to oven dryness with RMSEs less than 0.01. The GG model (Groenevelt and Grant, 2004) produced larger errors at the dry region, with significantly larger RMSEs and MEs than the FX model. Further evaluations indicated that when SWRC measurements in the 0–100 kPa suction range was applied for model establishment, the FX model was capable of producing acceptable SWRCs across the entire water content range. For a higher accuracy, the FX model requires soil water retention data at least in the 0- to 300-kPa range to extend the SWRC to oven dryness. Comparing with the Khlosi et al. (2006) model, which requires measurements in the 0–500 kPa range to reproduce the complete SWRCs, the FX model has the advantage of requiring less SWRC measurements. Thus the FX modeling approach has the potential to eliminate the processes for measuring soil water retention in the dry range.

## Introduction

Soil water retention curve (SWRC) describes the relationship between soil water content (θ) and matric suction (*h*). The information of SWRC is required in the simulation of water and solute transport in the soil [1]. Although various models have been proposed to describe the SWRC and most of the models perform well at high and medium water contents, they often fail to describe SWRC in the range beyond the wilting point [2], [3].

Several models have been applied to describe the SWRC from saturation to oven dryness [4]–[11]. Due to the difficulties in measuring SWRC at matric suction larger than 1500 kPa, however, much attention has been paid on extending the predictive capability of existing SWRC models to the dry region from limited measurements in the wet region. Some researchers (e.g., Schneider and Goss [3]) applied pedotransfer functions to obtain the van Genuchten [12] SWRC parameters using soil texture data, and then estimated the SWRC for the dry region using the Webb [9] model. Lu et al. [13] evaluated the performance of three extrapolative models (Fayer and Simmons, [8]; Webb, [9]; and Khlosi et al. [11]) on eight soils. When measurements in the 0–1500 kPa suction range were used, these models provided reliable SWRC results from saturation to oven-dryness. For the Khlosi et al. [11] model, data in the 0 to 500 kPa suction range were required to produce acceptable reliable results.

Fredlund and Xing [7] and Groenevelt and Grant [10] established SWRC models that are able to fit the measurements from saturation to oven dryness. It is unclear if the two models can be used to estimate the complete SWRC with limited measurements in the wet range. The objectives of this work are: (1) to compare the Fredlund and Xing [7] and Groenevelt and Grant [10] models in describing SWRC from saturation to oven dryness; and (2) to investigate if these models can be used to predict SWRC curve from saturation to oven dryness with limited measurements in the wet range.

## Materials and Methods

### Ethics Statement

The sampling locations are not privately-owned or protected in any way and the field activities did not involve endangered or protected species. No specific permissions were required for these sampling activities in this study.

### The Models

Based on the assumption that the shape of SWRC is dependent on soil pore-size distribution, Fredlund and Xing [7] proposed a general equation for the complete SWRC (hereafter the FX model):
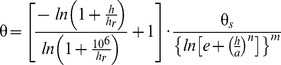
(1)


Where θ is the gravimetric soil water content (g g^−1^), *h* is the corresponding matric suction (kPa), *h_r_* is the suction corresponding to the residual water content that is generally taken as 3000 kPa [7], *a*, *n*, *m* are model parameters, and θ_s_ is the saturated soil water content (g g^−1^).

By anchoring the SWRC curve at zero water content with pF = 6.9, Groenevelt and Grant [10] presented the following model for the complete SWRC (hereafter the GG model):
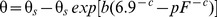
(2)where pF  =  log_10_ *h*, *h* is the matric suction in centimeters, and *b* and *c* are model parameters.

### SWRC data

The SWRC data from Lu et al. [13] are used in this study (Datasheet S1). The soil samples were collected from different areas of China and USA. Table 1 lists the basic characteristics of the eight soils. The pressure plate method was used to obtain SWRC data in matric suction range of 0–1500 kPa, and a Dewpoint Potential Meter (Model WP4-T, Decagon Device, Pullman, WA) was used to determine SWRC in the dry range (>1500 kPa). For more details of the soils and SWRC measurements, please refer to Lu et al. [13]. Additional data of 16 soils from Campbell and Shiozawa [5] and Prebble [14] were used to evaluate the extrapolative capacity of the SWRC models with reduced data sets (Datasheet S1). For convenience, the matric suction was expressed as pF value in the figures.

**Table 1:** Soil organic matter content (OM) and texture of the soils.

Soil ID	Texture	Sand	Silt	Clay	OM
		----------------------- % -----------------------			
1	Sand	93	1	6	0.07
2	Sandy loam	67	21	12	0.86
3	Loam	40	49	11	0.49
4	Silt loam	27	51	22	1.19
5	Silty clay loam	19	54	27	0.39
6	Silt loam	11	70	19	0.84
7	Silty clay loam	8	60	32	3.02
8	Silt loam	2	73	25	4.40

Soils 1-7 were collected in China and soil 8 was collected in the USA.

### Model Establishment and Evaluation

To obtain the model parameters, the mathematic software of Mathematica 7.0 (Wolfram, [15]) was applied for curve fitting. The nonlinear least-squares procedure was applied to minimize the sum of squared deviations of fitted data from measured data. The confidence intervals of model parameters were estimated to evaluate the extrapolative capacity of the SWRC models. Finally, the performances of the FX and GG models were evaluated with SWRC measurements by mean error (ME) and root mean square error (RMSE) of soil water content.

## Results and Discussion

### Model Performances when Established with Measurements in the 0-1500 kPa Range

**Figure 1:**
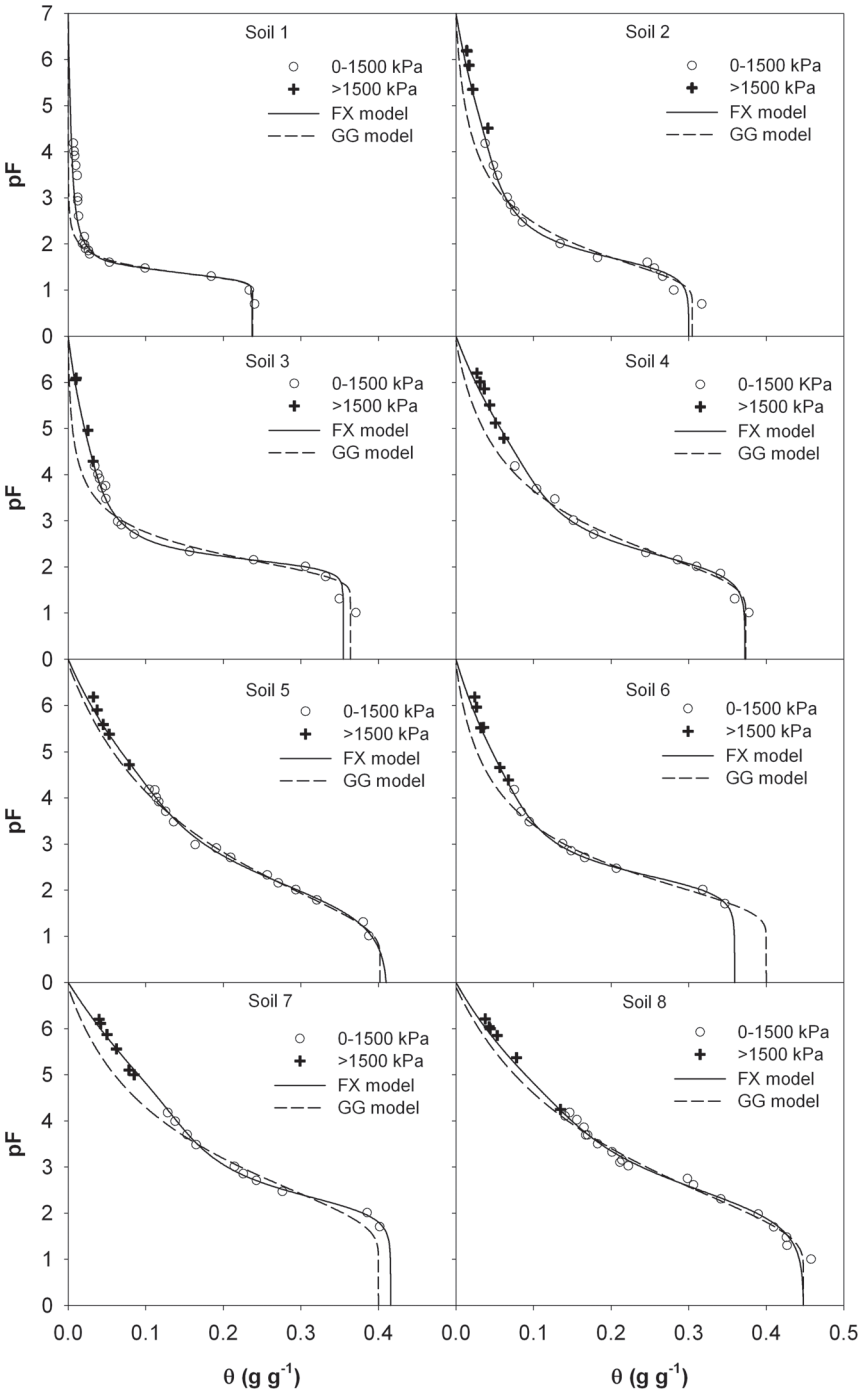
Comparison of measured and estimated soil water retention curves from the FX model (Fredlund and Xing, 1994) and the GG model (Groenevelt and Grant, 2004). The model parameters were obtained by fitting the models to the measurements in the suction range of 0 to 1500 kPa.

We first established the FX and GG models with SWRC measurements in the 0–1500 kPa suction range, and then applied the models to predict SWRCs at the dry region. Figure 1 presents the model results vs. measured data on eight soils from Lu et al. [13]. The RMSE and ME of the models are listed in Table 2. The results from the FX model agreed well with measured data with RMSE and ME less than 0.01, indicating that when measurements in the 0–1500 kPa range were applied for model establishment, the FX model could be used to extend the SWRC to oven dryness accurately.

**Table 2:** Mean error (ME) and root mean square error (RMSE) of the FX model (Fredlund and Xing, 1994) and the GG model (Groenevelt and Grant, 2004) for the tested soils from saturation to oven dryness.

	FX model		GG model	
Soil ID	RMSE	ME	RMSE	ME
1	0.004	−0.001	0.008	−0.005
2	0.009	−0.001	0.015	−0.005
3	0.006	−0.001	0.017	−0.007
4	0.006	−0.001	0.015	−0.008
5	0.005	−0.001	0.009	−0.003
6	0.004	−0.001	0.015	−0.008
7	0.005	0.000	0.020	−0.011
8	0.009	−0.002	0.013	−0.004

Soil water retention data in the 0–1500 kPa suction range were used for model establishment.

Compared to the FX model, the GG model gave mixed results on the eight soils. For soils 1 and 5, the GG model predictions agreed well with the experimental data. For the other soils, large prediction errors were observed (Table 2). For example, the GG model underestimated θ significantly on soils 3, 4, 6, and 7 in the higher suction region. On these four soils the RMSEs (>0.015) of the GG model were more than double that of the FX model.

Tables S1 and S2 summarize the model parameters and confidence limits with 95% confidence interval. A low confidence interval generally indicates a high accuracy in parameter estimation. For the GG model, the lower limits of parameter *b* had negative values on soils 6 and 7, indicating larger uncertainties in the estimated parameters. These results further confirm that the GG model fails to extrapolate the SWRC to oven dryness even the measurements in the 0–1500 kPa range are applied for model establishment.

### Evaluation of FX Model Established with Measurements in the 0–300 kPa and 0–100 kPa Ranges

**Figure 2:**
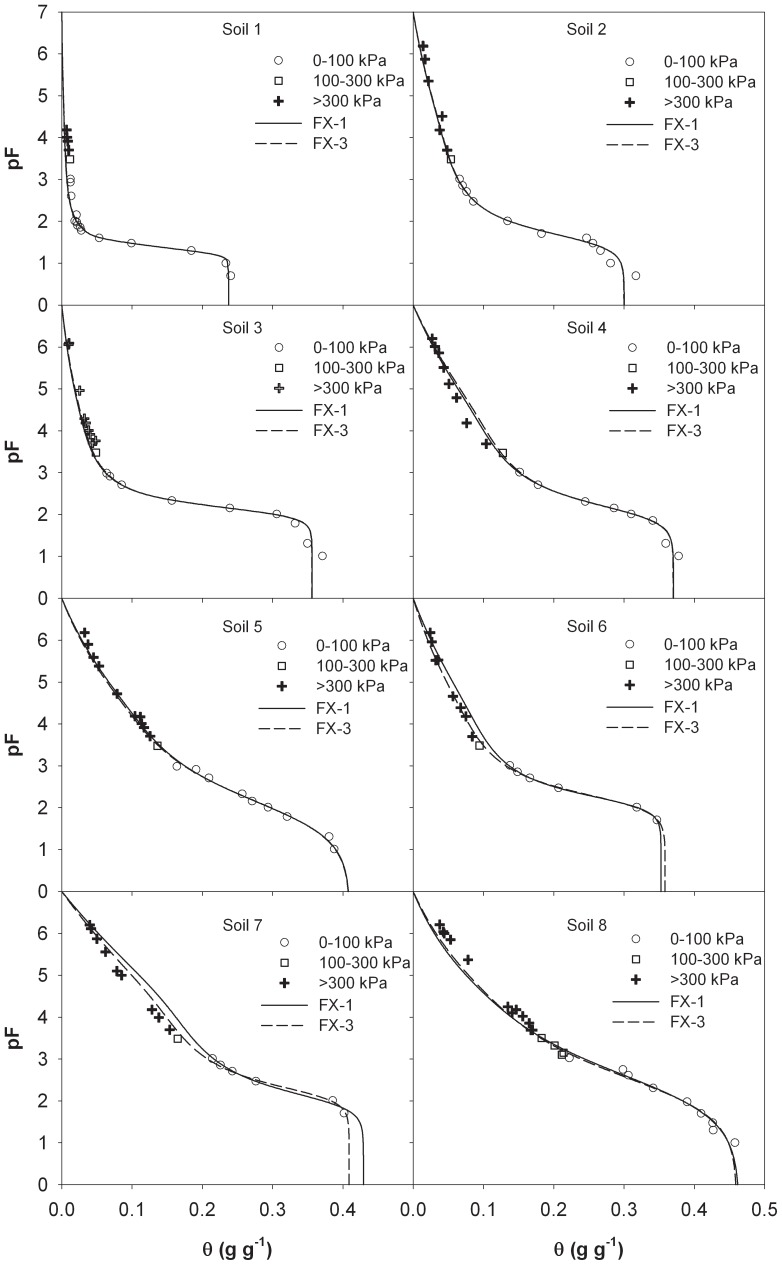
Comparison of measured and estimated soil water retention curves from oven-dryness to saturation from the FX model (Fredlund and Xing, 1994). FX-1 and FX-3 indicate that the parameters were obtained by fitting the models to the measurements in the suction range of 0 to 100 kPa and 0 to 300 kPa, respectively.

We further tested the prediction ability of the FX model in describing the entire SWRC when reduced data sets were used to obtain the model parameters. Due to the larger errors at the higher suction region, the GG model was not tested on these data sets. Figure 2 shows the calculated pF(θ) data from the FX model versus the measurements, where FX-3 and FX-1 represent models based on the 0–300 kPa and 0–100 kPa suction ranges, respectively.

When the model parameters were estimated from SWRC measurements in the 0–100 kPa suction range, the predictions from the FX model generally agreed with the experimental data (Figure 2), although a slightly larger but acceptable error was observed as compared with the results of Figure 1. Error analysis showed that on the eight soils from Lu et al. [13], the RMSEs of the FX-1 model were less than 0.016 (Table 3). Further analysis indicated that the FX-1 model performed better on soils with lower clay contents (Table 3). Similar results were also reported by Schneider and Goss [3] who showed that their pedotransfer function gave more accurate SWRC from saturation to oven dryness on coarse soils than on fine soils. Generally, the fine soils with larger clay contents have a wider range of “unknown dry region” where adsorbed water is dominant, and the prediction uncertainty of extrapolative models in the dry region of SWRC is increased.

**Table 3:** Mean error (ME) and root mean square error (RMSE) of the FX model (Fredlund and Xing, 1994) for the tested eight soils from saturation to oven dryness: FX-1 and FX-3 indicate that the parameters were obtained by fitting the models to the measurements in the suction range of 0 to 100 kPa and 0 to 300 kPa, respectively.

	FX-3 model		FX-1 model	
Soil ID	RMSE	ME	RMSE	ME
1	0.004	−0.001	0.004	−0.002
2	0.009	−0.001	0.009	−0.001
3	0.006	−0.002	0.007	−0.003
4	0.008	0.003	0.007	0.002
5	0.006	−0.003	0.006	−0.001
6	0.004	−0.001	0.007	0.004
7	0.008	0.004	0.016	0.011
8	0.012	−0.006	0.013	−0.005

When a larger suction range, e.g., SWRC data in 0–300 kPa, was used for model establishment, slight reductions in RMSEs and MEs were observed (Table 3). The confidence intervals of the FX-3 model parameters, however, were lower than that of the FX-1 model (Table S3), especially for soils 5, 7 and 8.

### Evaluation of FX Model Established with Reduced Data Sets from Literature

**Figure 3:**
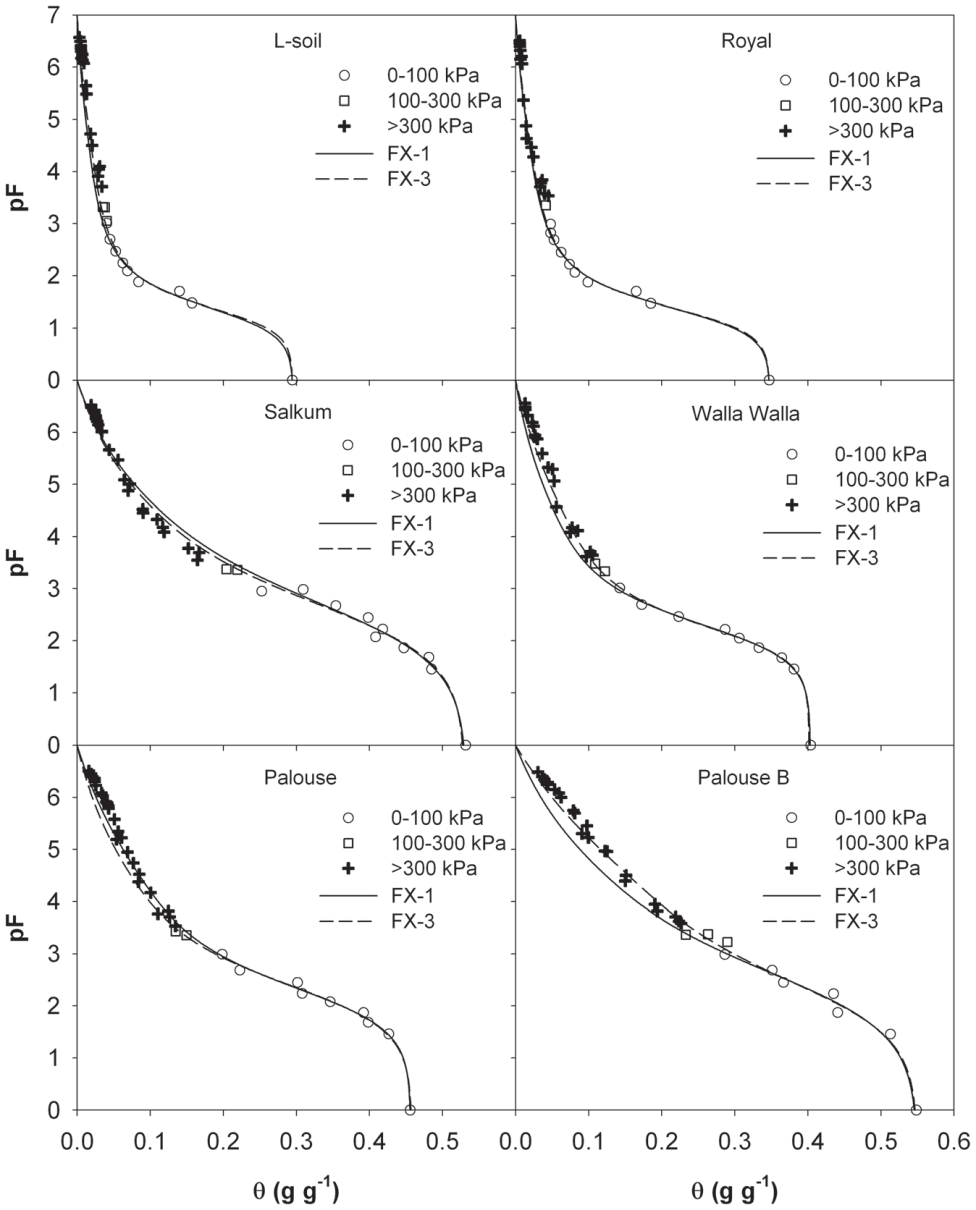
Comparison of measured and estimated soil water retention curves from oven-dryness to saturation from the FX model (Fredlund and Xing, 1994) on six soils measured by Campbell and Shiozawa (1992). FX-1 and FX-3 indicate that the parameters were obtained by fitting the models to the measurements in the suction range of 0 to 100 kPa and 0 to 300 kPa, respectively.

**Figure 4:**
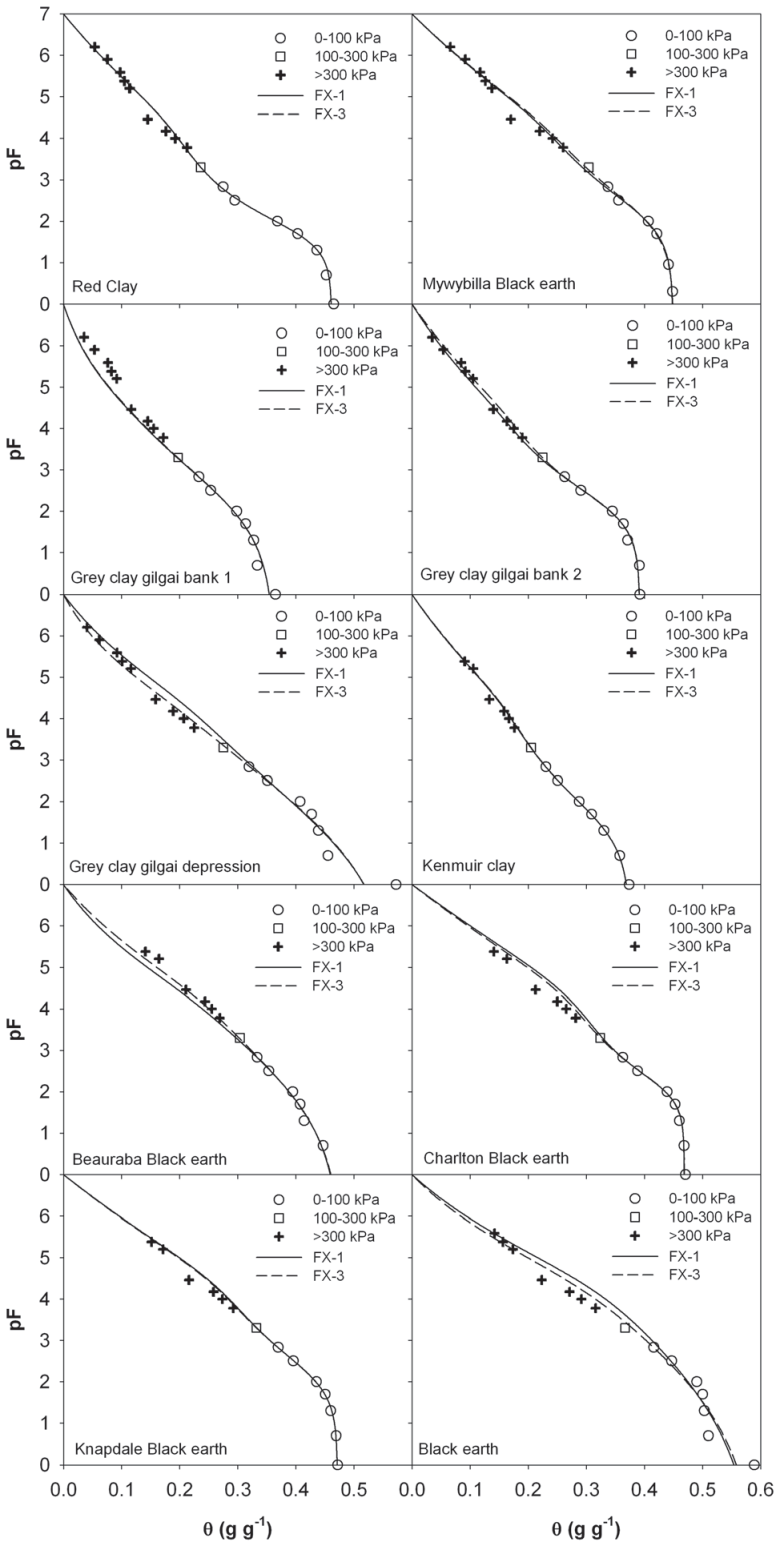
Comparison of measured and estimated soil water retention curves from oven-dryness to saturation from the FX model (Fredlund and Xing, 1994) on 10 soils from Prebble (1991). FX-1 and FX-3 indicate that the parameters were obtained by fitting the models to the measurements in the suction range of 0 to 100 kPa and 0 to 300 kPa, respectively.

Due to the scarce of SWRC data in the entire matric suction range, most extrapolative models are evaluated with data on six soils measured by Campbell and Shiozawa [5]. In this study, we further evaluated the performance of the FX model with 16 data sets: six soils from Campbell and Shiozawa [5] and 10 soils from Prebble [14]. Table 4 lists the details of the soils. Figure 3 shows the calculated pF(θ) data from the FX model versus the measurements on six soils of Campbell and Shiozawa [5]. It is evident that the FX-3 model provides accurate predictions in the entire water content range with RMSEs less than 0.015 (Table 5). When the model parameters are obtained from SWRC measurements in the 0–100 kPa suction range, the FX model also produces acceptable results (with RMSEs within 0.007–0.023). On the 10 soils from Prebble [14], the RMSEs of the FX-3 model are within 0.005–0.019 (Figure 4). Except for the Black Earth soil (with a RMSE of 0.027), the FX-1 model predictions generally agree with the measurements with RMSEs within 0.019 (Table 5). The large discrepancies between the measured and predicted values on the Black Earth are probably caused by errors in measurements, especially at near saturation region (Figure 4).

**Table 4:** Texture of the soils from Campbell and Shiozawa (1992) and Prebble (1991).

Soil ID	Texture	Sand	Silt	Clay
		---------- % ---------		
*Soils from Campbell and Shiozawa (1992)*				
L-soil	Sand	89	6	5
Royal	Sandy loam	54	31	15
Salkum	Silt loam	19	58	23
Walla Walla	Silt loam	23	63	14
Palouse	Silt loam	11	68	21
Palouse B	Silty clay	9	44	47
*Soils from Prebble (1991)*				
Red Clay	Clay	39	7	54
Mywybilla Black earth	Clay	32	14	54
Grey clay gilgai bank-1	Clay	33	18	49
Grey clay gilgai bank-2	Clay	31	16	53
Grey clay gilgai depression	Clay	25	16	59
Kenmuir clay	Sandy Clay Loam	57	17	26
Beauraba Black earth	Clay	23	16	61
Charlton Black earth	Clay	22	20	58
Knapdale Black earth	Clay	16	24	60
Black earth	Clay	23	18	59

**Table 5:** Mean error (ME) and root mean square error (RMSE) of the FX model (Fredlund and Xing, 1994) and KCGS model (Khlosi et al., 2006) for the tested 16 soils from Campbell and Shiozawa (1992) and Prebble (1991) from saturation to oven dryness: FX-1 and KCGS-1 indicate that the parameters were obtained by fitting the FX model and KCGS model to the measurements in the suction range of 0 to 100 kPa, respectively.

Soil ID	FX-1 model		KCGS-1 model		FX-3 model	
	RMSE	ME	RMSE	ME	RMSE	ME
*Soils from Campbell and Shiozawa (1992)*						
L soil	0.007	−0.003	0.022	0.000	0.006	−0.002
Royal	0.007	−0.003	0.027	0.000	0.007	−0.002
Salkum	0.018	0.006	0.014	−0.004	0.015	0.002
Walla Walla	0.011	−0.009	0.005	0.001	0.006	−0.003
Palouse	0.009	−0.002	0.010	0.006	0.012	−0.008
Palouse B	0.023	−0.018	0.028	−0.023	0.012	−0.002
*Soils from Prebble (1991)*						
Red Clay	0.008	0.002	0.029	−0.021	0.008	0.002
Mywybilla Black earth	0.011	0.002	0.017	−0.011	0.013	0.005
Grey clay gilgai bank-1	0.014	−0.009	0.012	−0.008	0.014	−0.009
Grey clay gilgai bank-2	0.005	−0.002	0.009	−0.005	0.005	0.002
Grey clay gilgai depression	0.019	0.010	0.015	−0.006	0.013	0.000
Kenmuir clay	0.004	0.001	0.025	−0.017	0.005	0.002
Beauraba Black earth	0.017	−0.011	0.029	−0.021	0.009	−0.004
Charlton Black earth	0.017	0.011	0.017	−0.012	0.013	0.008
Knapdale Black earth	0.011	0.006	0.021	−0.014	0.010	0.005
Black earth	0.027	0.015	0.023	−0.012	0.019	0.006

FX-3 indicates that the parameters were obtained by fitting the FX model to the measurements in the suction range of 0 to 300 kPa.

**Figure 5:**
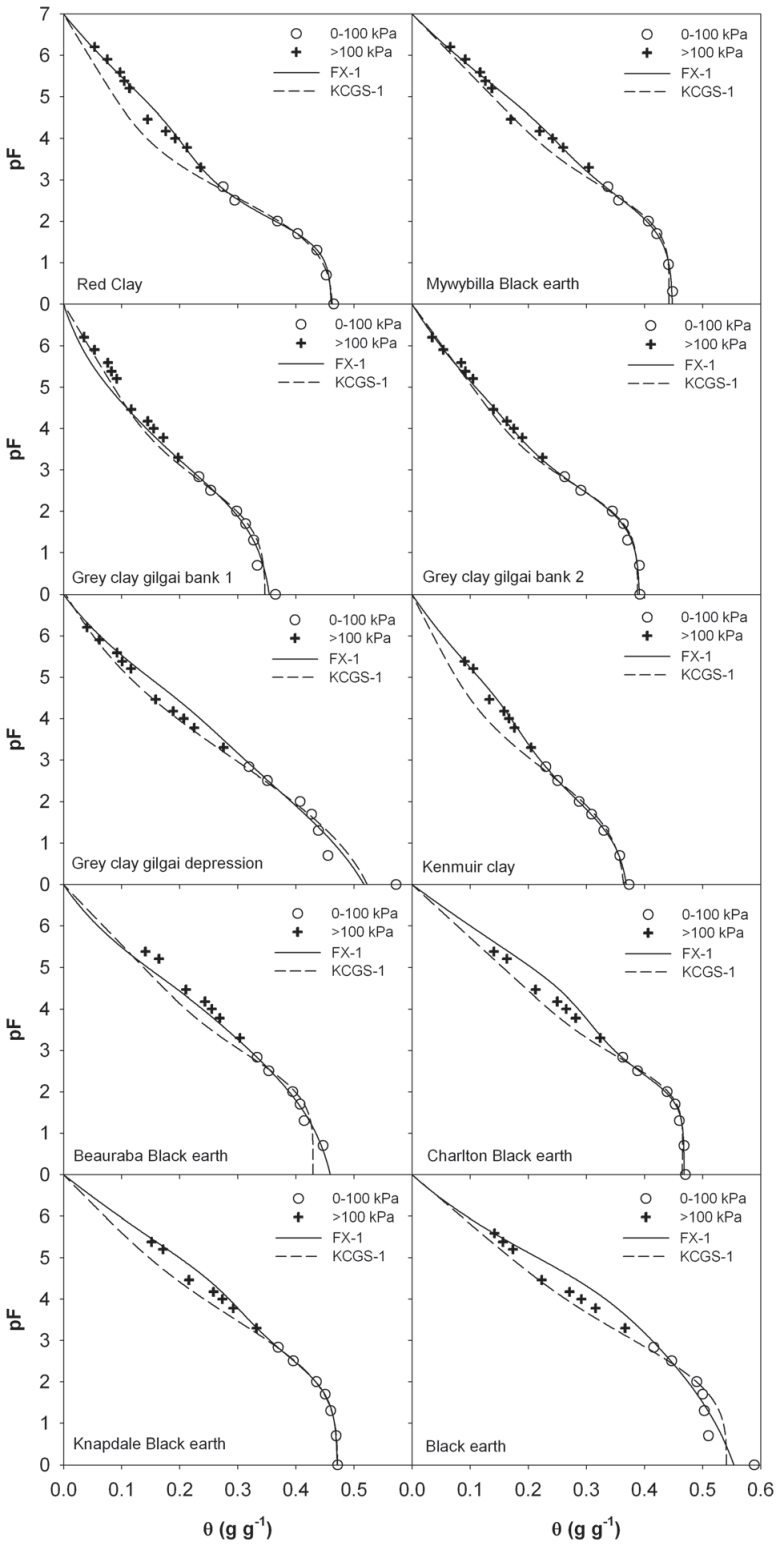
Comparison of measured and estimated soil water retention curves from oven-dryness to saturation from the FX model (Fredlund and Xing, 1994) and KCGS model (Khlosi et al., 2006) on 10 soils from Prebble (1991). FX-1 and KCGS-1 indicate that the parameters were obtained by fitting the FX model and KCGS model to the measurements in the suction range of 0 to 100 kPa, respectively.

It has been demonstrated that the Khlosi et al. [11] model is capable of producing the complete SWRCs from measurements in the 0–500 kPa range measurements (Lu et al. [13]). The proposed Khlosi et al. [11] function is
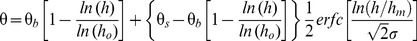
(3)where θ_s_ is the saturated water content, θ_b_ is a curve-fitting parameter representing the soil water content at *h* = 1, *h*_m_ is the matric suction corresponding to the median pore radius, σ is a curve-fitting parameter, and “erfc” is the complementary error function. Khlosi et al. (2006) tested the performance of their model with reduced data sets of soils from Campbell and Shiozawa (1992). Lu et al. (2008) evaluated the performance of the Khlosi et al. [11] model using reduced data sets of eight soils listed in Table 1. Here we further compared the extrapolative capability of the Khlosi et al. [11] model vs. the FX model using SWRC data of Prebble [14] in the 0–100 kPa suction range. Large deviations of Khlosi et al. [11] model in the dry regions were observed on the Red Clay, Kenmuir Clay, Beauraba Black earth, and Knapdale Black earth (Figure 5). For example, the RMSE of Khlosi et al. [11] model was 0.029 on Red Clay (Table 5), about four times that of the FX-1 model (0.008). Thus comparing with the Khlosi et al. [11] model, the FX model has the advantage of requiring less SWRC data.

## Conclusions

In this study, the extrapolation capacity of GG and FX model were evaluated on soils of various textures. When the model parameter was estimated with SWRC measurements in the 0–1500 kPa suction range, the results from the FX model agreed well with experimental results from saturation to oven dryness. The GG model performed well on some soils (soils 1 and 5), but produced larger errors in the other soils. The RMSEs of the FX model range from 0.004 to 0.009, considerably less than that of the GG model (0.008–0.020). Therefore, the GG model is not appropriate to extrapolate the SWRC to oven dryness under limited measurements.

When reduced data set in the 0–100 kPa suction range was used, the FX model was capable of producing SWRCs from saturation to oven dryness with acceptable accuracy. For greater prediction accuracy, SWRC measurements at least in the 0–300 kPa range were required for the FX model to estimate the complete SWRC accurately. With the FX approach, the needs for measuring soil water retention in the dry range can be eliminated.

## Supporting Information

Table S1 **Estimated model parameters and confidence limits for the FX model (Fredlund and Xing, 1994).** Soil water retention data in the 0-1500 kPa suction range were used for model establishment. The values in parentheses are the lower and upper limits of the 95% confidence interval.
(DOC)

Table S2 **Estimated model parameters and confidence limits for the GG model (Groenevelt and Grant, 2004).** The calculation was conducted on the measurement of soil water retention in the suction range of 0-1500 kPa. The values in parentheses are the lower and upper limits of the 95% confidence interval.
(DOC)

Table S3 **Estimated model parameters and confidence limits for the FX model (Fredlund and Xing, 1994).** FX-1 and FX-3 indicate that the parameters were obtained by fitting the models to the measurements in the suction range of 0 to 100 kPa and 0 to 300 kPa, respectively. The values in parentheses are the lower and upper limits of the 95% confidence interval.
(DOC)

Datasheet S1 **Soil water retention curve (SWRC) data from Lu et al. (2008), Campbell and Shiozawa (1992), and Prebble (1991).**
(XLS)
