# Supplementary material for: Extrapolative Capability of Two Models That Estimating Soil Water Retention Curve between Saturation and Oven Dryness
Source: PLoS One. 2014 Dec 2;9(12):e113518. doi: 10.1371/journal.pone.0113518 (PMC4252034; doi:10.1371/journal.pone.0113518)
Supplement: Table S1 — Estimated model parameters and confidence limits for the FX model (Fredlund and Xing, 1994). Soil water retention data in the 0-1500 kPa suction range were used for model establishment. The values in parentheses are the lower and upper limits of the 95% confidence interval. (DOC) [file pone.0113518.s001.doc]

**Table S1. Estimated model parameters and confidence limits for the FX model (Fredlund and Xing, 1994). Soil water retention data in the 0-1500 kPa suction range were used for model establishment. The values in parentheses are the lower and upper limits of the 95% confidence interval.**

| Soil ID | θ_s_ (g g^-1^) | *a* | *n* | *m* |
| --- | --- | --- | --- | --- |
| 1 | 0.24 (0.23, 0.24) | 2.09 (2.00, 2.19) | 5.74 (4.55, 6.92) | 1.08 (0.94, 1.22) |
| 2 | 0.30 (0.28, 0.32) | 3.21 (2.45, 3.96) | 2.46 (1.03, 3.90) | 0.72 (0.48, 0.96) |
| 3 | 0.35 (0.34, 0.37) | 11.14 (10.29, 11.98) | 4.11 (2.94, 5.27) | 0.76 (0.65, 0.87) |
| 4 | 0.37 (0.36, 0.39) | 9.77 (7.86, 11.68) | 1.92 (1.17, 2.67) | 0.61 (0.46, 0.76) |
| 5 | 0.41 (0.38, 0.44) | 4.91 (3.19, 6.64) | 1.00 (0.64, 1.36) | 0.74 (0.55, 0.94) |
| 6 | 0.36 (0.34, 0.38) | 12.34 (9.26, 15.43) | 2.23 (1.45, 3.00) | 0.65 (0.51, 0.79) |
| 7 | 0.42 (0.38, 0.45) | 12.24 (7.37, 17.11) | 2.16 (1.12, 3.21) | 0.46 (0.33, 0.59) |
| 8 | 0.45 (0.42, 0.47) | 12.48 (8.14, 16.81) | 1.15 (0.68, 1.62) | 0.65 (0.45, 0.85) |
