# Supplementary material for: Extrapolative Capability of Two Models That Estimating Soil Water Retention Curve between Saturation and Oven Dryness
Source: PLoS One. 2014 Dec 2;9(12):e113518. doi: 10.1371/journal.pone.0113518 (PMC4252034; doi:10.1371/journal.pone.0113518)
Supplement: Table S2 — Estimated model parameters and confidence limits for the GG model (Groenevelt and Grant, 2004). The calculation was conducted on the measurement of soil water retention in the suction range of 0-1500 kPa. The values in parentheses are the lower and upper limits of the 95% confidence interval. (DOC) [file pone.0113518.s002.doc]

**Table S2. Estimated model parameters and confidence limits for the GG model (Groenevelt and Grant, 2004).** The calculation was conducted on the measurement of soil water retention in the suction range of 0-1500 kPa. The values in parentheses are the lower and upper limits of the 95% confidence interval.

| Soil ID | θ_s_ (g g^-1^) | *b* | *c* |
| --- | --- | --- | --- |
| 1 | 0.24 (0.23, 0.25) | 9.14 (4.73, 13.55) | 7.18 (6.03, 8.32) |
| 2 | 0.30 (0.28, 0.33) | 4.34 (2.25, 6.42) | 2.56 (1.96, 3.17) |
| 3 | 0.36 (0.33, 0.39) | 37.87 (3.72, 72.02) | 4.69 (3.70, 5.69) |
| 4 | 0.37 (0.35, 0.40) | 10.16 (5.05, 15.27) | 2.52 (2.00, 3.04) |
| 5 | 0.40 (0.38, 0.43) | 4.22 (3.03, 5.41) | 1.44 (1.14, 1.73) |
| 6 | 0.40 (0.27, 0.53) | 9.38 (-5.09, 23.86) | 2.71 (1.52, 3.89) |
| 7 | 0.40 (0.30, 0.50) | 10.48 (-6.83, 27.79) | 2.16 (0.78, 3.55) |
| 8 | 0.45 (0.43, 0.47) | 6.84 (4.17, 9.50) | 1.71 (1.32, 2.10) |
