# Supplementary material for: Extrapolative Capability of Two Models That Estimating Soil Water Retention Curve between Saturation and Oven Dryness
Source: PLoS One. 2014 Dec 2;9(12):e113518. doi: 10.1371/journal.pone.0113518 (PMC4252034; doi:10.1371/journal.pone.0113518)
Supplement: Table S3 — Estimated model parameters and confidence limits for the FX model (Fredlund and Xing, 1994). FX-1 and FX-3 indicate that the parameters were obtained by fitting the models to the measurements in the suction range of 0 to 100 kPa and 0 to 300 kPa, respectively. The values in parentheses are the lower and upper limits of the 95% confidence interval. (DOC) [file pone.0113518.s003.doc]

**Table S3. Estimated model parameters and confidence limits for the FX model (Fredlund and Xing, 1994). FX-1 and FX-3 indicate that the parameters were obtained by fitting the models to the measurements in the suction range of 0 to 100 kPa and 0 to 300 kPa, respectively. The values in parentheses are the lower and upper limits of the 95% confidence interval.**

| Soil ID | θ_s_ (g g^-1^) | *a* | *n* | *m* |
| --- | --- | --- | --- | --- |
|  |  | FX-1 model | |  |
| 1 | 0.24 (0.23, 0.24) | 2.12 (2.02, 2.22) | 5.38 (4.21, 6.54) | 1.15 (0.98, 1.32) |
| 2 | 0.30 (0.27, 0.33) | 3.21 (2.20, 4.22) | 2.44 (0.42, 4.47) | 0.73 (0.33, 1.12) |
| 3 | 0.36 (0.34, 0.37) | 11.35 (9.75, 12.95) | 3.66 (1.84, 5.47) | 0.84 (0.55, 1.13) |
| 4 | 0.37 (0.36, 0.38) | 9.40 (7.33, 11.46) | 2.25 (1.04, 3.46) | 0.53 (0.31, 0.76) |
| 5 | 0.41 (0.33, 0.49) | 5.22 (1.23, 9.22) | 0.99 (-0.28, 2.26) | 0.75 (-0.24, 1.75) |
| 6 | 0.35 (0.34, 0.36) | 11.89 (10.61, 13.16) | 2.83 (2.20, 3.47) | 0.52 (0.44, 0.61) |
| 7 | 0.43 (0.26, 0.60) | 8.23 (-5.46, 21.92) | 2.47 (-5.69, 10.62) | 0.37 (-0.48, 1.22) |
| 8 | 0.46 (0.37, 0.56) | 15.00 (-47.60, 77.60) | 0.83 (-0.72, 2.38) | 0.91 (-1.97, 3.78) |
|  |  | FX-3 model | |  |
| 1 | 0.24 (0.23, 0.24) | 2.11 (2.01, 2.22) | 5.49 (4.26, 6.73) | 1.13 (0.96, 1.30) |
| 2 | 0.30 (0.27, 0.33) | 3.21 (2.32, 4.09) | 2.49 (0.69, 4.29) | 0.71 (0.40, 1.03) |
| 3 | 0.36 (0.34, 0.37) | 11.28 (9.97, 12.59) | 3.77 (2.18, 5.37) | 0.82 (0.60, 1.04) |
| 4 | 0.37 (0.36, 0.38) | 9.22 (7.75, 10.68) | 2.38 (1.44, 3.33) | 0.51 (0.37, 0.64) |
| 5 | 0.41 (0.36, 0.46) | 5.55 (2.63, 8.47) | 0.97 (0.22, 1.71) | 0.79 (0.21, 1.36) |
| 6 | 0.36 (0.33, 0.39) | 12.26 (8.22, 16.30) | 2.29 (1.02, 3.55) | 0.63 (0.39, 0.87) |
| 7 | 0.41 (0.37, 0.45) | 12.54 (5.54, 19.54) | 2.76 (0.17, 5.35) | 0.39 (0.17, 0.60) |
| 8 | 0.46 (0.41, 0.51) | 13.00 (1.86, 24.14) | 0.94 (0.19, 1.68) | 0.81 (0.08, 1.53) |
